# Three-lncRNA signature is a potential prognostic biomarker for pancreatic adenocarcinoma

**DOI:** 10.18632/oncotarget.24443

**Published:** 2018-02-08

**Authors:** Xiuhui Shi, Yan Zhao, Ruizhi He, Min Zhou, Shutao Pan, Shuo Yu, Yu Xie, Xu Li, Min Wang, Xingjun Guo, Renyi Qin

**Affiliations:** ^1^ Department of Biliary-Pancreatic Surgery, Affiliated Tongji Hospital, Tongji Medical College, Huazhong University of Science and Technology, Wuhan, China

**Keywords:** long noncoding RNA, pancreatic adenocarcinoma, TCGA, ceRNA, prognosis

## Abstract

Pancreatic adenocarcinoma (PAAD) is a highly aggressive and metastatic cancer characterized by poor survival rates. Long non-coding RNAs (lncRNAs) play important roles in various biological processes, including cancer and PAAD. To identify the specific lncRNAs associated with PAAD and analyze their function, we constructed a global triple network based on the competitive endogenous RNA (ceRNA) theory and RNA-seq data from The Cancer Genome Atlas. Using 182 PAAD cases, we established a lncRNA–miRNA–mRNA co-expression network, which was composed of 43 lncRNA nodes, 253 mRNA nodes, 11 miRNA nodes, and 475 edges. Six lncRNAs in the ceRNA network were closely related to overall survival, and a three-lncRNA signature predicted survival of PAAD patients. Protein–protein interaction network data revealed that five genes were associated with overall survival. These results provide novel insight into the function of a lncRNA-associated ceRNA network in the pathogenesis of PAAD, and indicate that the identified three-lncRNA signature may serve as an independent prognostic marker in PAAD.

## INTRODUCTION

Pancreatic adenocarcinoma (PAAD) and ductal adenocarcinoma, the most common and deadly forms of human pancreatic cancer, are highly invasive and metastatic, and are associated with poor survival [1]. Due to the aggressive nature of PAAD and development of chemoresistance, PAAD is the fourth leading cause of cancer-related mortality in the western world, with an overall 5-year survival rate around 4% [2, 3]. Recent advances in genomic cancer research have led to numerous biomarker discoveries and improvements in patient care. However, in PAAD, specific biomarkers are still urgently needed.

Long noncoding RNAs (lncRNAs) are RNA transcripts that are longer than 200 nt and exhibit limited or no protein-coding capacity. Many lncRNAs are uniquely expressed in differentiated tissues or specific cancer types [4–6]. LncRNAs drive many important cancer phenotypes by interacting with other cellular molecules, including DNA, RNA, and proteins [7, 8]. Recent studies have demonstrated that lncRNAs are associated with the pathogenesis of different diseases, including PAAD [9–11]. Compared to protein-coding genes, lncRNAs have exhibited a superior potential as diagnostic and prognostic biomarkers. For example, lncRNAs have been used as diagnostic and prognostic biomarkers in hepatocellular carcinoma, gastric cancer, ovarian cancer, and prostate cancer [12–15].

Although thousands of lncRNAs have been discovered and recorded in public databases, such as NONCODE, LNCipedia, and LncRNADB, the functional characterization of lncRNAs is still in its infancy; up to now, only few lncRNAs have been functionally characterized [16, 17]. It has been suggested that functionally related lncRNAs are associated with functionally related mRNAs or miRNAs, and involved in similar diseases. This association has been demonstrated in several diseases, but has not been studied in PAAD [18–20].

In this study, we have constructed a lncRNA-related network using the data from The Cancer Genome Atlas (TCGA), and the recently developed competitive endogenous RNA (ceRNA) theory [21].

## RESULTS

### Identification of differentially expressed RNAs in PAAD

**Figure 1:**
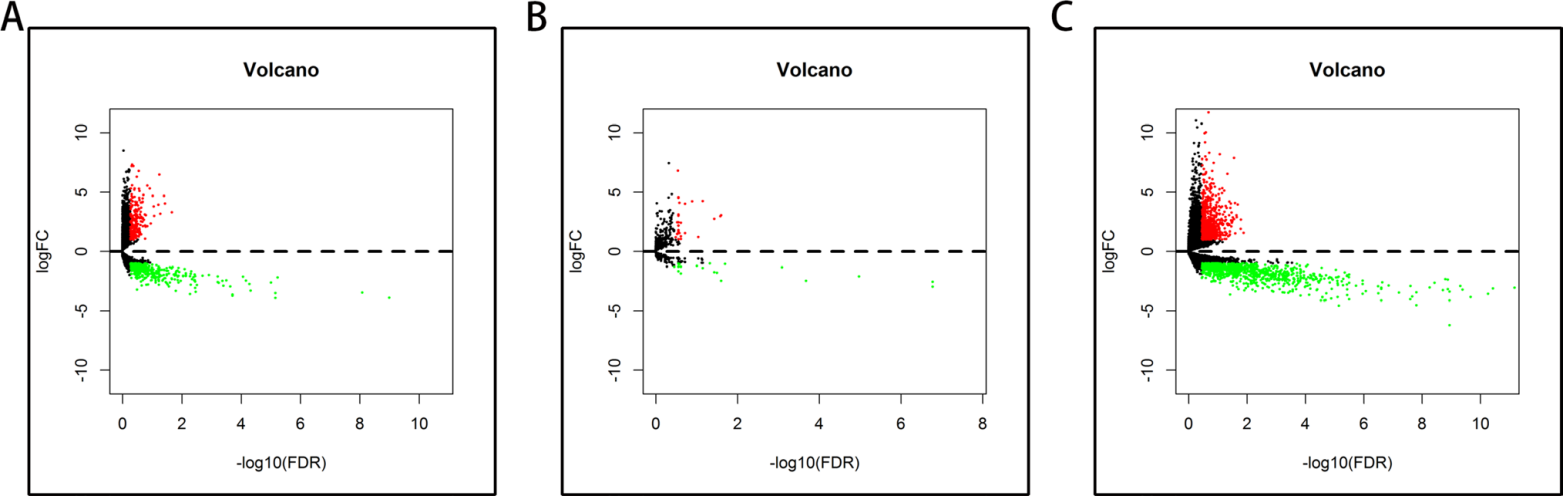
Volcano plot of the differentially expressed lncRNAs, miRNAs, and mRNAs between pancreatic adenocarcinoma and control tissues Red dots indicate high expression and green dots indicate low expression of lncRNAs, miRNAs, and mRNAs. Black dots show the lncRNAs with expression of Fold change <2. The X axis represents an adjusted FDR and the Y axis represents the value of log2FC. (**A**) Differentially expressed lncRNAs; (**B**) Differentially expressed miRNAs; (**C**) Differentially expressed mRNAs.

**Figure 2:**
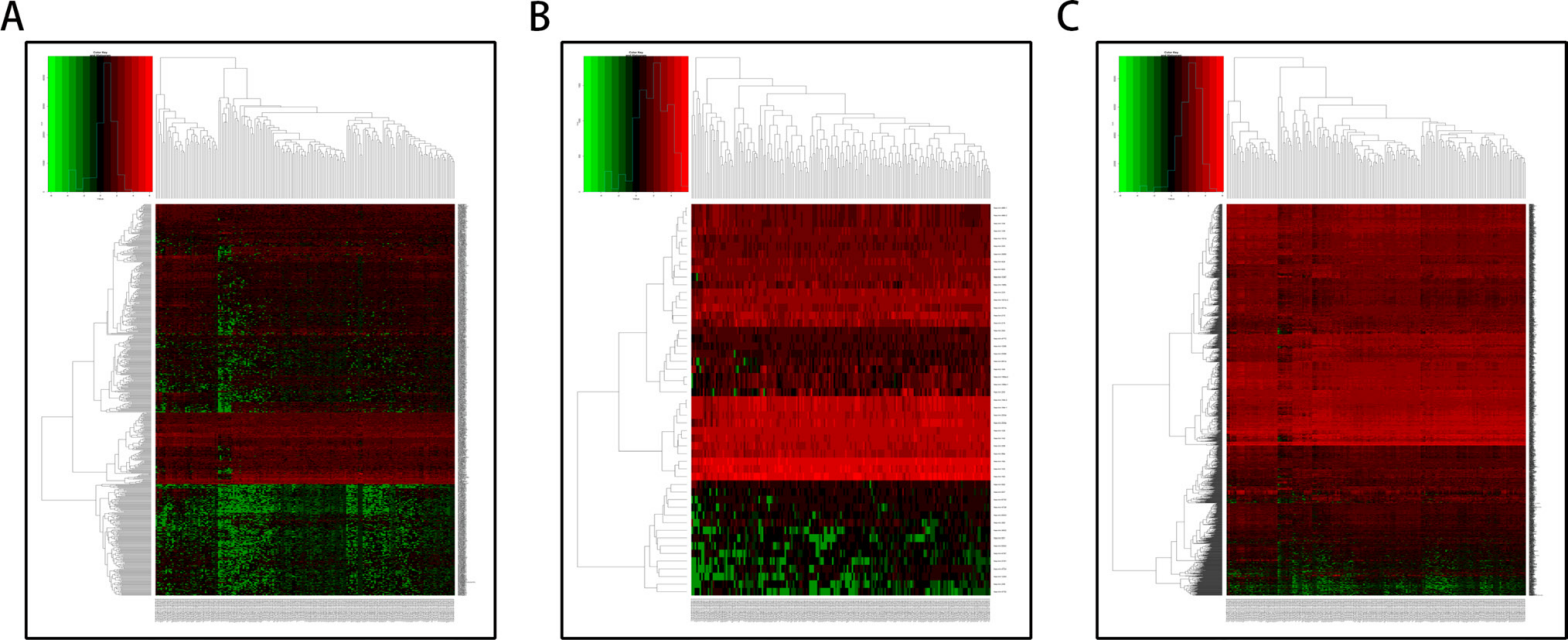
Hierarchical clustering of pancreatic adenocarcinoma and control tissues by differentially expressed RNAs The heat-map consists of 4 normal tissues (left part) and 178 pancreatic adenocarcinoma tissues (right part). Each row represents an RNA level, and each column represents a sample. Red stands for high expression and green indicates low expression of lncRNAs, miRNAs, or mRNAs. (**A**) Differentially expressed lncRNAs; (**B**) Differentially expressed miRNAs; (**C**) Differentially expressed mRNAs.

A total of 182 samples were analyzed in this study, including 178 pancreatic adenocarcinoma tissues and 4 matched normal tissues. Using the cut-off criteria (*P* < 0.05 and |Fold change| > 2.0), 650 differentially expressed lncRNAs (DELs), 50 differentially expressed miRNAs (DEMis), and 1724 differentially expressed mRNAs (DEMs) were identified between pancreatic adenocarcinoma tissues and matched normal tissues (Supplementary Tables 1–3). Altogether, 220 over-expressed and 430 under-expressed lncRNAs, 29 over-expressed and 21 under-expressed miRNAs, and 643 over-expressed and 1081 under-expressed mRNAs were identified. In order to verify the *P*-value and fold change using a different test, we used the volcano plot (Figure 1). Unsupervised hierarchic cluster analysis revealed that pancreatic adenocarcinoma tissues could be distinguished from normal tissues based on differentially expressed RNAs patterns (Figure 2).

### Construction of lncRNA–miRNA–mRNA network

**Figure 3:**
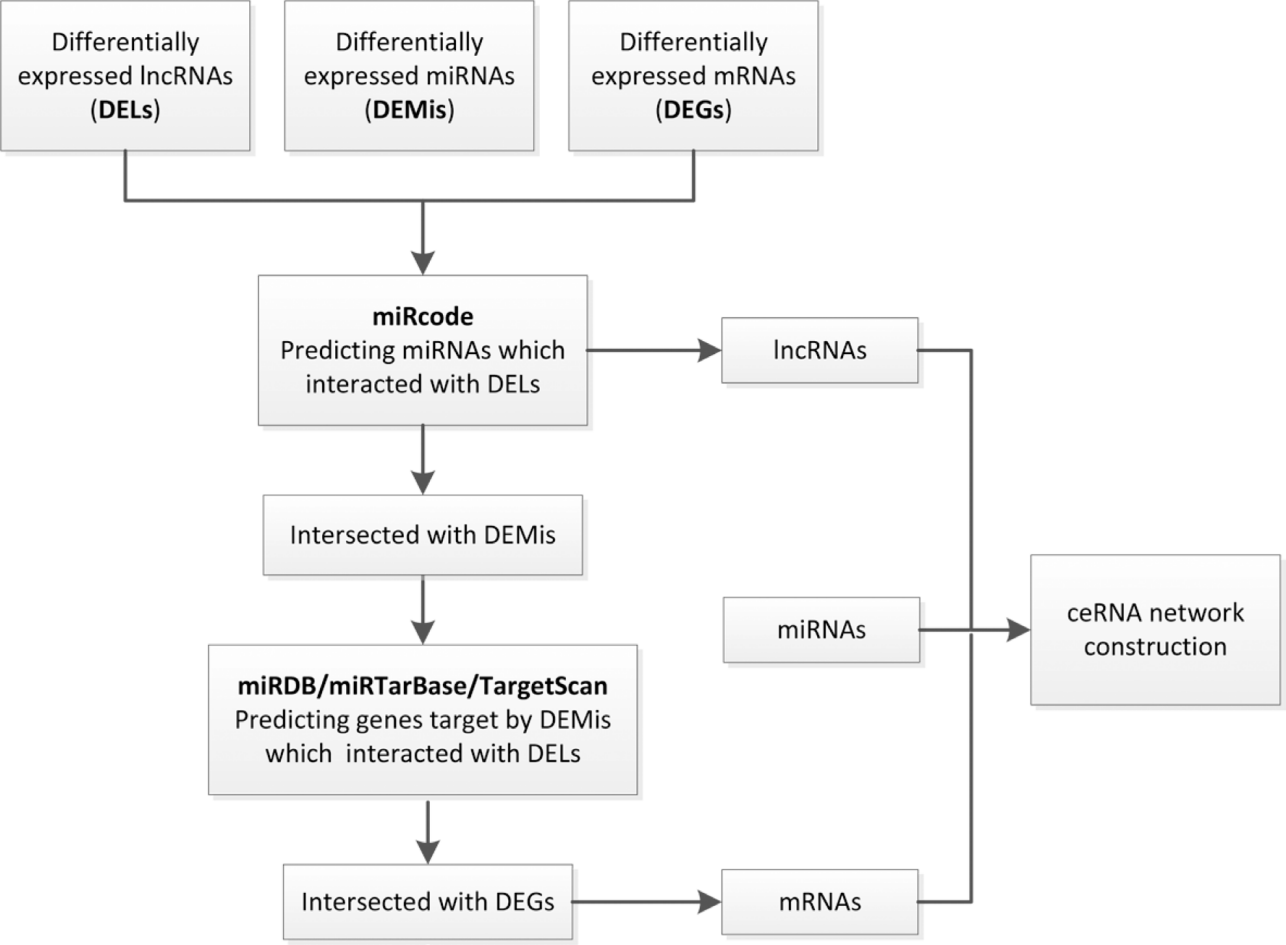
A flowchart of ceRNA network construction (i) DELs, DEMis and DEMs with fold change > 2.0 and *P*-value < 0.05 were used; (ii) miRNA-lncRNA interactions were predicted by miRcode; (iii) lncRNAs not associated with DEMis were removed; (iv) mRNAs targeted by miRNAs were identified using miRDB, miRTarBase and TargetScan databases; (v) mRNAs that were not DEMs were removed; (vi) ceRNA network was constructed.

**Figure 4:**
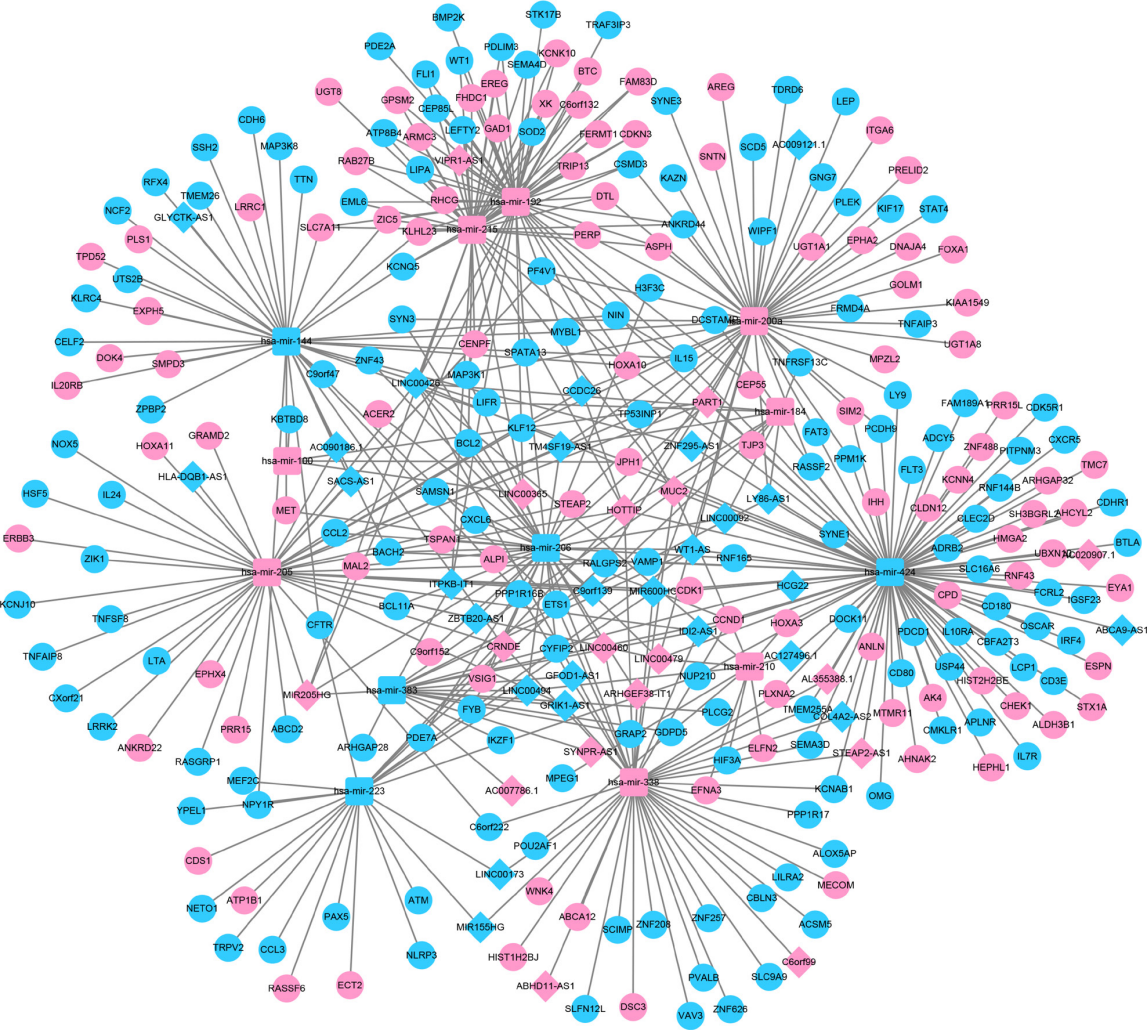
Illustration of the lncRNA–miRNA–mRNA network The rhombus represents lncRNAs, the circle represents mRNAs, and the square represents miRNAs. There were 43 lncRNAs, 13 miRNAs, 253 mRNAs and 475 edges in the network.

In order to establish lncRNA–miRNA–mRNA ceRNA network, lncRNAs and mRNAs targeted by miRNAs were identified from the above data. The flow chart for ceRNA network construction is shown in Figure 3. The results showed that 43 specific lncRNAs interacted with 13 specific miRNAs (Supplementary Table 4). Target mRNAs of the 13 miRNAs were predicted using the TargetScan, miRDB, and miRTarBase databases. A total of 3715 target mRNAs of 13 miRNAs were identified Supplementary Table 5. Among the 3715 target mRNAs, 253 mRNAs were identified as DEMs. To elucidate the functions of the lncRNAs acting as miRNA targets, a network of lncRNAs, miRNAs, and mRNAs was constructed and visualized with Cytoscape. As shown in Figure 4, the lncRNA–miRNA–mRNA network was composed of 43 lncRNA nodes, 13 miRNA nodes, 253 mRNA nodes, and 475 edges.

### Identification of survival-related lncRNAs in PAAD

**Figure 5:**
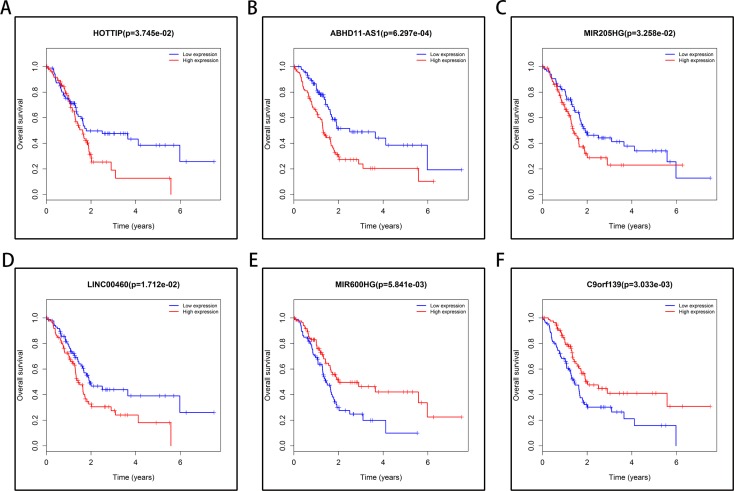
Six lncRNAs were associated with overall survival in 177 PAAD patients by using Kaplan–Meier curve and Log-rank test The patients were stratified into high level group and low level group according to median of each lncRNA. (**A**) HOPPIT; (**B**) ABHD11-AS1; (**C**) MIR205HG; (**D**) LINC00460; (**E**) MIR600HG; (**F**) C9orf139.

To identify the lncRNAs associated with an overall survival (OS) in PAAD, we evaluated the association between lncRNAs expression and OS in 177 PAAD patients using Kaplan–Meier curve and Log-rank test. The results showed that four lncRNAs (HOPPIT, ABHD11-AS1, MIR205HG, and LINC00460) negatively correlated with OS, and two miRNAs (MIR600HG and C9orf139) positively correlated with OS (Figure 5).

### Prognostic value of six lncRNAs risk score in PAAD

**Figure 6:**
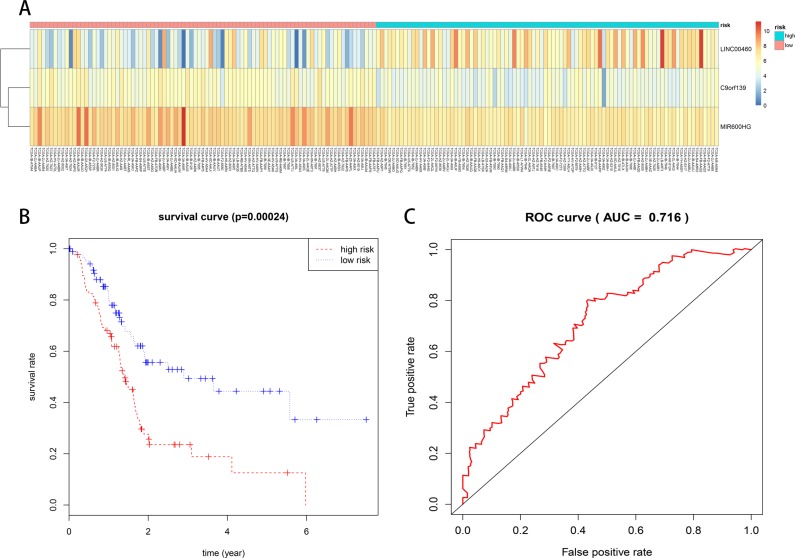
Prognostic evaluation of the 3-lncRNA signature in PAAD patients (**A**) Expression heat-map of 3 prognostic lncRNAs. (**B**) Kaplan–Meier survival curve analysis for overall survival of PAAD patients using the 3-lncRNA signature. (**C**) ROC curve analysis of the 3-lncRNA signature.

We constructed a prognostic signature by integrating the expression profiles of 6 lncRNAs and corresponding estimated regression coefficients. Then, we calculated risk scores for each patient, and ranked them according to increased scores. Using multivariate Cox regression analysis, our results indicated that a three-lncRNA (LINC00460, C9orf139, and MIR600HG) signature might provide a powerful information for the prognosis of PAAD patients. Expression of the three prognostic lncRNAs is shown in the heat-map (Figure 6A). A total of 177 patients with intact survival information were classified into a high risk group (*n* = 88) and a low risk group (*n* = 89) according to the median risk scores. Survival analysis was performed using the Kaplan–Meier method with a Log-rank statistical test. The results showed that patients in the high risk group had significantly worse OS than patients in the low risk group (Figure 6B). Receiver operating characteristic (ROC) curve was used to test the effect of the three-lncRNA signature (high risk vs. low risk) on OS (Figure 6C).

### Functional implication of prognostic lncRNA signature using enrichment analysis

**Figure 7:**
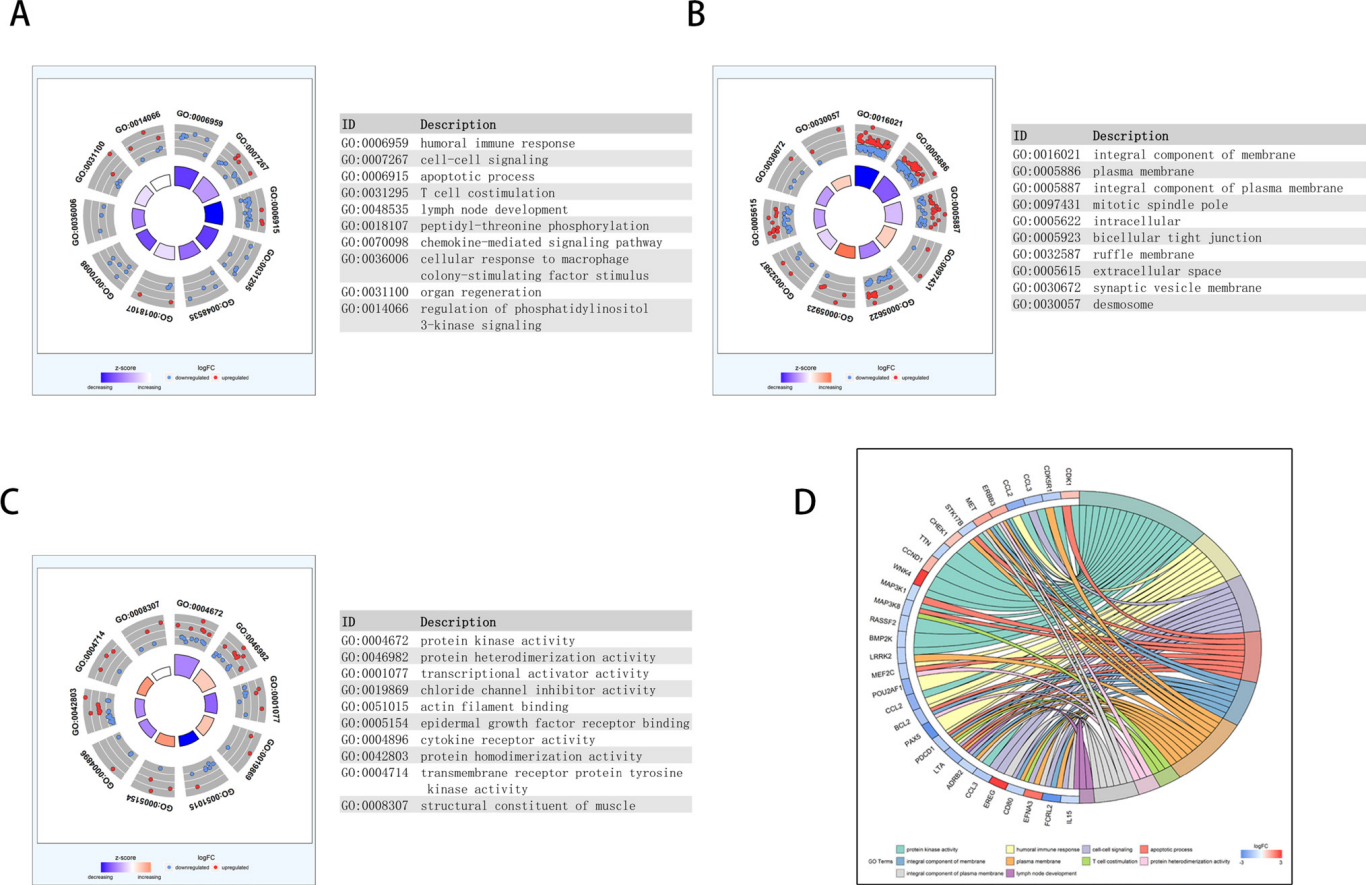
Functional analysis of key lncRNAs in PAAD The outer circle shows a scatter plot of the expression (logFC) of differentially expressed mRNAs in each enriched gene ontology (GO) term: red circles indicate upregulation and blue circles indicate downregulation. The inner ring is a bar plot where the height of the bar indicates the significance of GO terms (log10-adjusted *P* value), and color corresponds to the z-score: blue, decreased; red, increased; and white, unchanged.(**A**) Biological process (BP); (**B**) CC; (**C**) MF. (**D**) The plot shows the relationship between statistically significant top 30 mRNAs and their related GO terms; for each gene, the logFC value is shown by red/blue colored rectangles.

**Figure 8:**
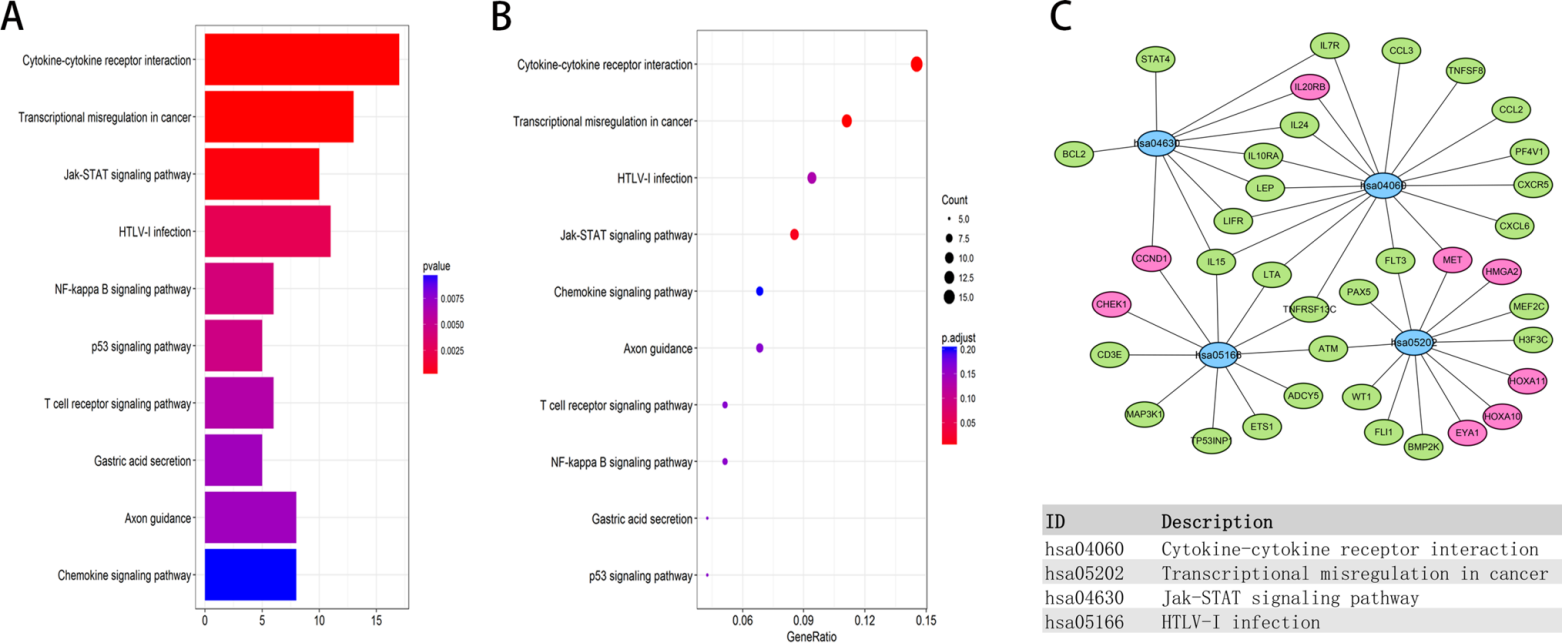
KEGG pathway functional enrichment analysis of lncRNA-related DEMs in the network (**A**, **B**) Top ten pathways of lncRNA-related DEMs. (**C**) Gene-concept networks by KEGG analysis of differentially expressed mRNAs, using Cytoscape. Blue hubs correspond to the most enriched KEGG pathway; red and green nodes represent upregulation and downregulation of mRNAs, respectively.

We performed enrichment analyses to elucidate the biological function of co-expressed DEMs of the lncRNAs in the network. Gene ontology (GO) analysis revealed that 70 enriched clusters were associated with biological processes (BP), 12 with cellular components (CC), and 20 with molecular function (MF). The top ten enriched clusters are shown in Figure 7A–7C. The first enriched biological process was humoral immune response. The first enriched cellular component and molecular function were integral component of membrane and protein kinase activity, respectively. Figure 7D displays the relationship between statistically significant top 30 DEMs and their related GO terms. In addition, a total of 42 KEGG pathways were enriched. The first enriched KEGG pathway was the Cytokine-cytokine receptor interaction signaling pathway. The top ten enriched functional analyses are shown in Figure 8A and 8B. The top 4 functional enrichment analyses of the DEMs network are shown in Figure 8C.

### Functional implication of prognostic lncRNA signature using PPI network construction

**Figure 9:**
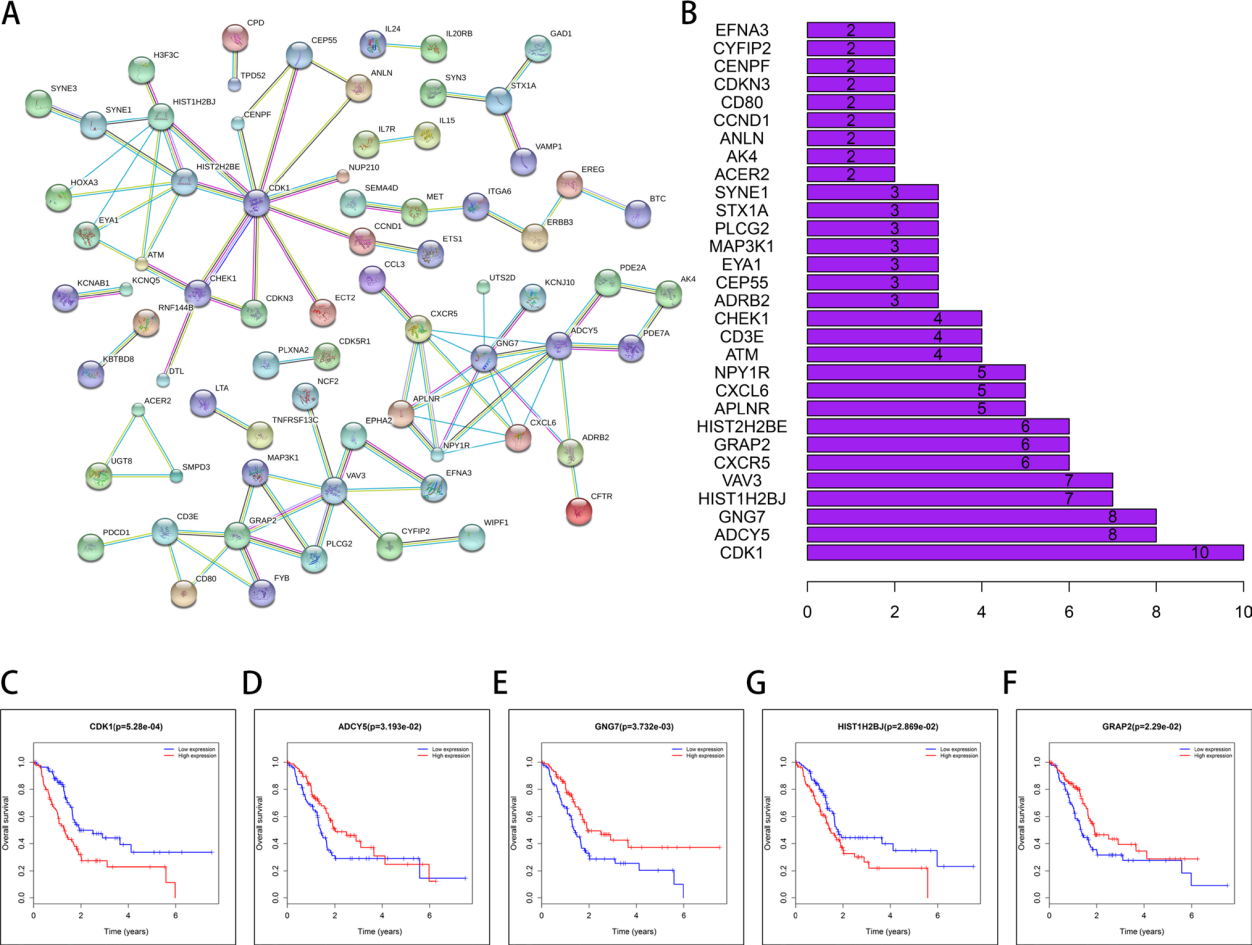
STRING database analysis of the PPI network of DEMs (**A**, **B**) CDK1, ADCY5, GNG7, HIS1T1H2BJ, VAV3, CXCR5, GRAP2 and HIST2H2H2BE show increased association with other node proteins (more than 5), indicating that they have higher hub degrees. The patients were stratified into high level group and low level group according to median of each lncRNA. (**C**) CDK1; (**D**) ADCY5; (**E**) GNG7; (**F**) HIS1T1H2BJ; (**G**) GRAP2.

STRING (a database of known and predicted protein interactions) was used to predict protein–protein interactions (PPI) among the DEMs. First, the 253 DEMs in the network were submitted to the STRING website. Then, the obtained PPI data with combined scores greater than 0.900 were selected for constructing PPI networks and disconnected nodes in the network were hidden. In the PPI networks, 8 node proteins, CDK1, ADCY5, GNG7, HIST1H2BJ, VAV3, CXCR5, GRAP2 and HIST2H2BE, showed a strong association with other node proteins (more than 5), indicating that they have higher hub degrees (Figure 9A, 9B). Among the 8 hub genes, 5 genes (CDK1, ADCY5, GNG7, HIST1H2BJ, and GRAP2) were associated with OS, indicating that they might play important roles in the PAAD.

## DISCUSSION

Recent advances in genomic cancer research have led to numerous biomarker discoveries and improvements in patient care. For example, Oncotype DX, a 21-gene signature, has been used to predict estrogen receptor positive breast cancer recurrence in patients with lymph node negative disease; patients with high risk scores are recommended for adjuvant chemotherapy [22]. While there have been successful stories of biomarker application for clinical use in breast cancer [22], lung cancer [23] and melanoma [24], sadly, the clinical application of biomarkers in PAAD is very limited.

Aberrant expression of lncRNAs has been widely observed in various diseases, and dysregulated lncRNAs have emerged as key regulators of vital biological functions in cancer cells [25, 26]. However, only a few studies have described lncRNA profiles in PAAD by RNA-seq data analysis [27]. Song *et al.* [28] have constructed lncRNA-mRNA network by using dysregulated lncRNAs and mRNAs, and the ceRNA hypothesis. To date, only a few studies have investigated the interaction between lncRNAs and mRNAs, or lncRNAs and miRNAs in PAAD. Results from those studies have indicated that lncRNAs play a key role in competitive endogenous RNA (ceRNA) networks, but such ceRNA networks are still poorly characterized [29, 30]. The ceRNA hypothesis has been proposed as a novel regulatory mechanism functioning through miRNA competition [31]. Recent studies of ceRNA cross-talks have indicated that ceRNAs are regulated by miRNAs, which interact with lncRNAs in complex ceRNA networks [32].

In the present study, we identified lncRNAs, miRNAs and mRNAs differentially expressed in PAAD from TCGA database. Then, we constructed the PAAD-specific lncRNA–miRNA–mRNA ceRNA network, which provides an important insight about the key RNAs of the ceRNA-mediated gene regulatory network in the initiation and development of PAAD. We analyzed OS of PAAD patients using 43 differentially expressed lncRNAs in the network; six of the lncRNAs were associated with OS in PAAD patients. After multivariate Cox regression analysis, the three-lncRNA (LINC00460, miR205HG and c3orf133) signature was established and identified as an independent prognostic factor for PAAD patients. ROC curve was used to test the effect of this three-lncRNA signature on OS.

Gene ontology (GO) analysis indicated that the aberrantly expressed genes mainly function in metabolism and immune response. Based on KEGG pathway analysis, several cancer-related pathways were detected, including p53 signaling pathway, JAK-STAT pathway, and T cell receptor signaling pathway. Hu *et al.* [33] reported that fenofibrate inhibited pancreatic cancer cell proliferation via accumulation of p53 protein and activation of p53 pathway mediated by upregulation of lncRNA MEG3. Denley *et al.* [34] found that activation of the IL-6R/Jak/Stat pathway was associated with a poor outcome in resected pancreatic ductal adenocarcinoma. In addition, it was reported that the T cell receptor signaling pathway was associated with the PAAD pathogenesis [35, 36]. Through PPI network construction, we found that several proteins formed the network, including CDK1, ADCY5, GNG7, HIST1H2BJ, VAV3, CXCR5, GRAP2 and HIST2H2BE; 5 of these hub genes (CDK1, ADCY5, GNG7, HIST1H2BJ and GRAP2) were associated with OS, indicating importance of this ceRNA network in PAAD.

Recent studies have indicated that lncRNAs function as crucial components of ceRNA networks by modulating other RNA transcripts [19, 37]. For example, HOTAIR may act as an endogenous sink by binding miR-331-3p, thereby abolishing the miRNA- inhibitory activity and introducing an additional level of post-transcriptional regulation [38]. Hence, a potential connection between lncRNA, miRNA and mRNA may exist during the PAAD pathogenesis. In the present study, we constructed the lncRNA–miRNA–mRNA ceRNA network to reveal a novel ceRNA regulatory network in PAAD. With respect to the associations between 43 cancer specific lncRNAs from ceRNA network and patient survival, we found that 6 lncRNAs were related to OS, and three of them might serve as prognostic biomarkers for PAAD patients. Among these 3 lncRNAs, only LINC00460 has been reported in the regulatory networks in carcinoma; the other lncRNAs (MIR600HG and C3orf139) have not been reported [39]. Furthermore, a recent study has shown that LINC00460 is up-regulated in several cancers, including pancreatic cancer, breast cancer, lung cancer, colon cancer and bladder cancer [40], indicating that LINC00460 may play an important role in PAAD. Several cancer specific lncRNAs identified in the above ceRNA network, such as PART1 [41], LINC00356 [42], CCDC26 [43], and HOTTIP [44–46] have been identified as potential diagnostic and prognostic cancer biomarkers. In addition, WT-AS [47], LINC00092 [48], ABHD11-AS1 [49], and MIR205HG [50] have been associated with cancer initiation and progression.

In conclusion, we have searched for PAAD-specific lncRNAs by using a large scale analysis of hundreds of candidate lncRNAs in TCGA database, and identified aberrant expression profiles of cancer specific lncRNAs that correlated with OS. The identified PAAD-specific lncRNAs might serve as potential biomarkers in PAAD. Importantly, we have constructed the lncRNA–miRNA–mRNA ceRNA network to evaluate the ceRNA function in PAAD. We have found that three lncRNAs are differentially expressed in PAAD, and associated with an overall survival in PAAD patients. While efficacy of a single marker has been limited, multi-markers based models may provide a better prognostic information. Finally, we have constructed the three-lncRNA signature that correlates with PAAD patient survival, indicating that it may serve as an independent prognostic marker in PAAD.

## MATERIALS AND METHODS

### RNA sequence data collection and processing

The raw sequencing data and clinical information were downloaded from TCGA database (https://cancergenome.nih.gov/). The inclusion criteria were set as follows: (1) the sample included both miRNA sequencing data and clinical information; (2) the sample included prognosis information. A total of 182 samples were analyzed in this study, including 178 pancreatic adenocarcinoma tissues and 4 matched normal tissues. The RNA sequencing data were processed using R language package. The fold change (FC) values of individual RNA levels were calculated; differentially expressed RNAs with |FC| > 2.0 and *P*-value < 0.05 were considered to be significant.

### Construction of lncRNA–miRNA–mRNA ceRNA network

The ceRNA network was constructed based on the relationship between lncRNA, miRNA, and mRNA. To construct the ceRNA network, differentially expressed lncRNAs, miRNAs, and mRNAs with |FC| > 2.0 and *P*-value < 0.05 were used. The miRcode database [51] was used to predict the lncRNA-miRNA interactions. The miRDB [52], Targetscan [53], and miRTarBase [54] databases were used to predict the mRNAs targeted by miRNAs. Furthermore, the predicted miRNAs and differentially expressed data of TCGA were combined to select the interacting lncRNAs and mRNAs. Cytoscape [55] was used to construct and visualize the lncRNA–miRNA–mRNA ceRNA network.

### Analysis of lncRNAs in the network and patient prognosis

The differentially expressed lncRNA profiles were normalized by log2 transformation. The prognostic value of each differentially expressed lncRNA was evaluated using Kaplan–Meier curve and Log-rank method. The lncRNAs associated with overall survival (OS) were identified as candidates of prognostic lncRNA signature, and subjected to multivariate Cox regression analysis. Using the lncRNA signature, PAAD patients were classified into high risk and low risk groups using the median risk score. Differences in OS between the high risk and low risk groups were evaluated by Kaplan–Meier method. Receiver operating characteristic (ROC) curve was used to test the effect of the lncRNA signature (high risk vs. low risk) on OS. The area under the curve (AUC) under binomial exact confidence interval was calculated to generate the ROC curve.

### Functional enrichment analysis

Using annotation, visualization, and integrated discovery (DAVID) database, the functional enrichment analyses of 253 differentially expressed mRNAs (DEMs) in the network, and gene ontology (GO) function analysis were carried out. Kyoto Encyclopedia of Genes and Genomes (KEGG) pathway enrichment analysis was carried out for those 253 DEMs in the network using the database of KOBAS [56]. In the GO analysis, the categories included biological process (BP), cellular component (CC), and molecular function (MF), and *P*-value < 0.05 was regarded as statistically significant. To add quantitative molecular information to GO of interest, we used GOCircle and GOChord plot functions of GOplot R package [57], which permits to incorporate data derived from expression analysis with those obtained from the functional annotation enrichment analysis. In the KEGG pathways analysis, enriched pathways were identified according to the hypergeometric distribution with a *P*-value < 0.01, and were analyzed using the cluster Profiler package [57]. In addition, to provide a readable graphic representation of the complex relationships between DEMs and relative KEGG pathway, the “pathway-gene network” was constructed by Cytoscape.

### Protein–protein interaction analysis by STRING

Protein products of the 253 DEMs in the network were analyzed by the online tool, STRING [58], to predict protein-protein interactions. A combined score of not < 0.9 (highest confidence score) was considered significant. The hub protein was selected based on its association with other proteins. DEMs associated with other DEMs indicated corresponding protein–protein interactions (PPI).

### Statistical analysis

Statistical analysis was performed using the R 3.4.1 software. The data were expressed as mean ± standard deviation (SD), and analyzed using the paired *t*-test. The significance level was set as 0.001 as a default to control the false discovery rate (FDR). *P*-value < 0.05 was considered statistically significant.

## SUPPLEMENTARY MATERIALS TABLES












