# Supplementary material for: Three-lncRNA signature is a potential prognostic biomarker for pancreatic adenocarcinoma
Source: Oncotarget. 2018 Feb 8;9(36):24248–59. doi: 10.18632/oncotarget.24443 (PMC5966255; doi:10.18632/oncotarget.24443)
Supplement: Supplementary file 1 [file oncotarget-09-24248-s001.pdf]

Three-lncRNA signature is a potential prognostic biomarker for pancreatic adenocarcinoma

SUPPLEMENTARY MATERIALS

Supplementary Table 1: Differentially Expressed LncRNAs. See Supplementary_Table_1

Supplementary Table 2: Differentially Expressed MicroRNAs. See Supplementary_Table_2

Supplementary Table 3: Differentially Expressed mRNAs. See Supplementary_Table_3

Supplementary Table 4: MiRNAs targeted by 43 lncRNAs. See Supplementary_Table_4

Supplementary Table 5: mRNAs targeted by 13 miRNAs. See Supplementary_Table_5
